# Performance of TaqMan array card to detect TB drug resistance on direct specimens

**DOI:** 10.1371/journal.pone.0177167

**Published:** 2017-05-04

**Authors:** Sayera Banu, Suporn Pholwat, Suporn Foongladda, Rattapha Chinli, Duangjai Boonlert, Sara Sabrina Ferdous, S. M. Mazidur Rahman, Arfatur Rahman, Shahriar Ahmed, Eric R. Houpt

**Affiliations:** 1 Mycobacteriology Laboratory, Infectious Diseases Division, International Center for Diarrhoeal Diseases Research, Dhaka, Bangladesh; 2 Division of Infectious Diseases and International Health, Department of Medicine, University of Virginia, Charlottesville, Virginia, United States of America; 3 Department of Microbiology, Faculty of Medicine Siriraj Hospital, Mahidol University, Bangkok, Thailand; The University of Hong Kong, CHINA

## Abstract

Culture based phenotypic drug susceptibility testing (DST) for *Mycobacterium tuberculosis* (TB) is time consuming therefore rapid genotypic methods are increasingly being utilized. We previously developed and evaluated on TB isolates a rapid genotypic TaqMan array card (TAC) that detects mutations in several resistance-associated genes using dozens of primer pairs, probes, and high resolution melt analysis, with >96% accuracy versus Sanger sequencing. In this study we examined the performance of TAC on sputum, comparing results between 71 paired sputum and TB isolates of which 62 were MDR-TB. We also adapted the TAC to include wild-type probes and broadened coverage for *rpoB* and *gyrA* mutations. TAC was 89% successful at detecting wild-type or mutations within *inhA*, *katG*, *rpoB*, *eis*, *gyrA*, *rplC*, and *pncA* on smear positive sputa and 33% successful on smear negative sputa. The overall accuracy of these detections as compared to the TAC results of the paired isolate was 95% ± 7 (average sensitivity 98% ± 3; specificity 92% ± 14). Accuracy of sputum TAC results versus phenotypic DST for isoniazid, rifampin, ofloxacin/moxifloxacin, and pyrazinamide was 85% ± 12. This was similar to that of the isolate TAC results (accuracy 88% ± 13), thus inaccuracies primarily reflected intrinsic genotypic-phenotypic discordance. The TAC is a rapid, modular, comprehensive, and accurate TB DST for the major first and second line TB drugs and could be used for supplemental testing of GeneXpert resistant smear positive sputum.

## Introduction

Phenotypic drug susceptibility test (DST) for *Mycobacterium tuberculosis* is time consuming therefore molecular diagnostics are becoming widely utilized [1]. Commercial kits including the GenoType MTBDR*plus*, GenoType MTBDR*sl* (Hain Lifescience GmbH, Nehren, Germany) and GeneXpert MTB/RIF (Cepheid, Sunnyvale, USA) can detect drug resistance on direct specimens and cultured isolates [2–5]. The GeneXpert MTB/RIF, and the newer MTB/RIF Ultra, are limited to detecting rifampin resistance (RIF). The MTBDR*plus* can detect resistance to RIF plus isoniazid (INH), while the MTBDR*sl* version 2.0 detects resistance to injectable aminoglycosides (amikacin/kanamycin), and fluoroquinolones (ofloxacin/moxifloxacin). However the exact sequence mutation is not known, pyrazinamide is not tested, and the line probe methodology requires multiple steps including amplification, hybridization, and washing. We previously developed and evaluated a rapid genotypic TaqMan array card (TAC) method, which is a customizable 384 well card that runs 8 samples and compartmentalizes each sample into 48 different quantitative PCR reactions. We initially devised the method to detect the commonest resistance-associated mutations for rifampin, isoniazid, injectable aminoglycosides, fluoroquinolones, ethambutol, and streptomycin via sequence-specific probes, and to interrogate *pncA* sequence by high resolution melt analysis to obtain a pyrazinamide result. The assay was evaluated on 328 isolates and provided greater than 96% fidelity versus Sanger sequencing or the Hain Line probe result [6, 7].

In this work we modified the TAC into a version 2 which includes not only mutation but also wild-type probes, added several new emerging *rpoB* mutations to better capture rifampin resistance, included genotypic assays to detect linezolid resistance, and most importantly evaluated the performance of the assay on 71 direct sputum samples.

## Materials and methods

### Specificity testing of the assays

The specificity of the assays included in this TAC version 2 was evaluated on 384 well plates on the ViiA7 platform (Applied Biosystems, Life Technologies Corporation, Carlsbad, CA, USA). Assays were tested with wild-type H37Rv, previously-characterized TB isolates with mutations at *inhA*-8C, -15T, *katG*315Thr, *rpoB*511Pro, 513Glu, 513Leu, 516Val, 516Tyr, 526Tyr, 526Asp, 526Leu, 531Leu, 531Trp, 533Pro, *rrs*1401G, 1484T, *eis*-14T, -10A, *gyrA*90Val, 94Gly, 94Tyr, 94Ala, 94Asn, and synthetic plasmid controls (Genewiz Inc., South Plainfield, NJ, USA) including p*rpoB*513Lys, p*eis*-12T, p*rplC*154Arg, p*23S*2447T, and p*23S*2576T. Primer/probe sets (0.05μl of each forward and reverse primer, 0.02μl of each probe of 50μM stock, final concentrations of 0.5μM and 0.2μM, respectively) were assayed in a 5μl PCR mixture containing 2.5μl of 2x MeltDoctor HRM master mix (Applied Biosystems, Life Technologies Corporation, Carlsbad, CA, USA), 0.1 μl of 2.5 μM ROX reference dye, 1.28μl nuclease free water, and 1μl (100 pg/μl) of genomic DNA. Cycling conditions included an initial denaturation at 95°C for 10 min, followed by 40 cycles of denaturation at 95°C for 15 sec and annealing/extension at 64°C for 1 min.

### Clinical samples and culture conditions

The 71 samples were collected during 2015–2016 and included 38 MDR-TB, 2 non-MDR-TB, and 7 susceptible strain from MDR/XDR TB surveillance projects among high MDR risk patients in Bangladesh (International Centre for Diarrhoeal Diseases and Research, Dhaka, Bangladesh) and 24 consecutive MDR specimens from a large clinical microbiology laboratory in Thailand (Department of Microbiology, Faculty of Medicine Siriraj Hospital, Mahidol University, Bangkok, Thailand). We selected this sample size to obtain at least 10 fluoroquinolone and pyrazinamide resistant isolates. Sputum samples were processed for acid fast bacilli (AFB) staining and digestion-decontamination, sediments were inoculated into Lowenstein-Jensen and MGIT (Mycobacteria Growth Indicator Tube, Becton Dickinson, Sparks, MD) media for culture at 37°C up to 8 and 6 weeks, respectively, and 500 μl aliquot sediments were stored at -80°C until use. Positive cultures were identified as *M*. *tuberculosis* complex using in-house real-time PCR for Thailand samples, and GeneXpert MTB/RIF for Bangladesh samples. All work was reviewed and approved by Institutional Biosafety and Human Investigation Committees at the University of Virginia, International Centre for Diarrhoeal Diseases and Research, Bangladesh and Mahidol University, Thailand. Written consent was obtained from all participants except for Thailand where deidentified isolates were used and therefore consent was waived by the ethics committee.

### Phenotypic drug susceptibility testing

*M*. *tuberculosis* isolates underwent susceptibility testing per local laboratory practices in Thailand via agar proportion on Middlebrook 7H10 media with critical concentration recommended by CLSI [8] for isoniazid (INH; 0.2 μg/ml), rifampin (RIF; 1 μg/ml), amikacin (AMK; 4 μg/ml), kanamycin (KAN; 5 μg/ml), ofloxacin (OFX; 2 μg/ml), and moxifloxacin (MXF; 0.5 μg/ml). Bangladesh used the Lowenstein-Jensen proportion method with critical concentration 0.2 μg/ml (INH), 40 μg/ml (RIF), 30 μg/ml (KAN), and 2 μg/ml (OFX). The pyrazinamide (PZA) and linezolid (LZD) susceptibility test were performed by MGIT960 (Mycobacteria Growth Indicator Tube, Becton Dickinson, Sparks, MD) with critical concentration 100 μg/ml and 1 μg/ml respectively.

### Genotypic testing

**Fig 1:**
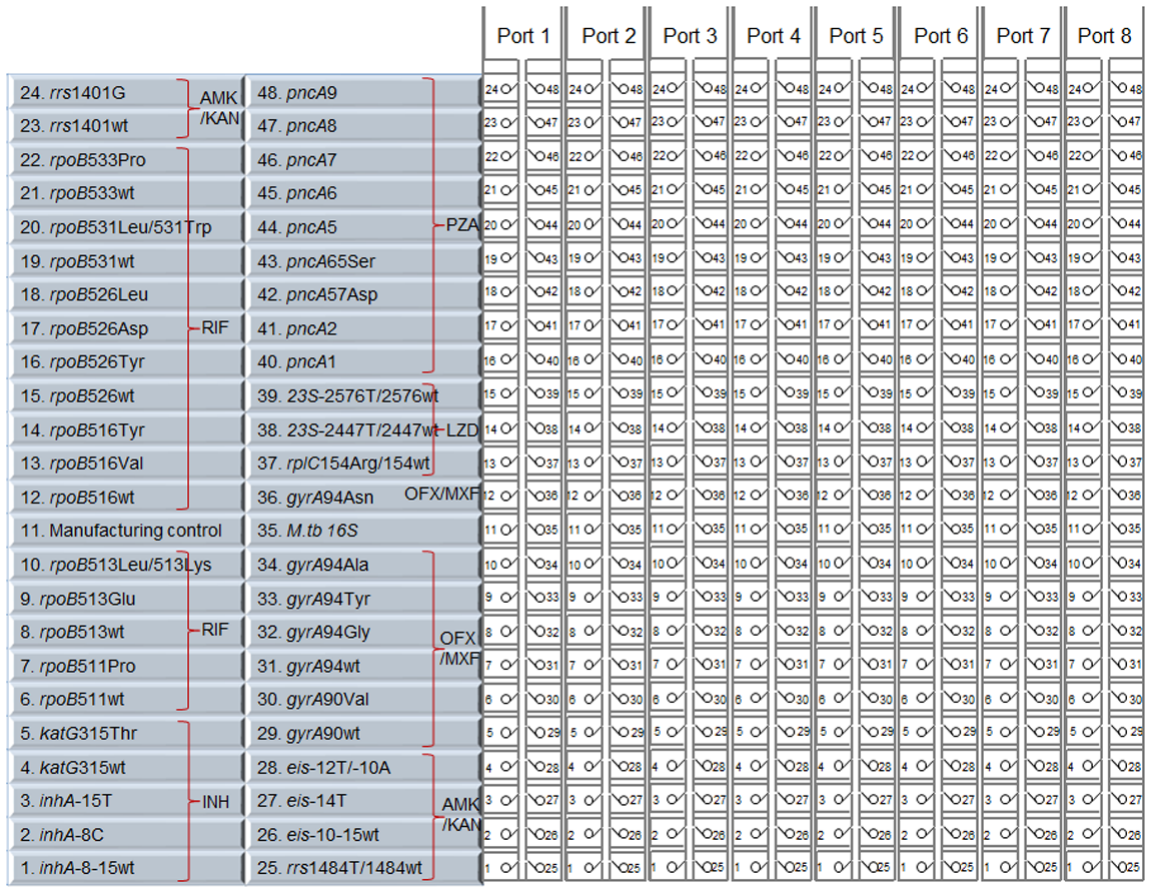
TB drug resistance TAC version 2. TAC tests 8 samples and compartmentalizes 48 assays per sample. Assays are grouped according to drug isoniazid (INH), rifampin (RIF), amikacin (AMK), kanamycin (KAN), ofloxacin (OFX), moxifloxacin (MXF), linezolid (LZD), and pyrazinamide (PZA). Each assay is shown on the basis of gene nucleotide or codon. Wt = wild-type. A/B indicates duplex assays.

When MDR-TB isolates were identified, we retrieved their preceding sputum sediment, and the sputum-isolate pair underwent DNA extraction (DNeasy Blood and Tissue kit, Qiagen Inc., Valencia, CA, USA) following the protocol described previously [9]. The paired sputum and isolate were characterized genotypically using a TAC [6] version 2. We incorporated wild-type probes for each target and included additional resistance-associated probes for *rpoB* and *gyrA* to now interrogate all the major (>3% sensitivity) mutations described by the TB Drug Resistance Database [10] or ReseqTB.org. We also included the major *eis* mutations described by Georghiou *et al* [11] and included assays for *rplC* and *23S* rRNA for linezolid (LZD). To accommodate these changes, we excluded ethambutol and streptomycin-specific assays given that DST for these drugs is rarely utilized by MDR TB programs. We also removed *gyrB* assays, for which there are no recognized drug-resistance mutations according to either of the above-mentioned databases. In total we include 11 primer sets and 44 sequence-specific probes for detection of the major resistance-associated mutations of the *inhA* and *katG* (for INH), *rpoB* (for RIF), *rrs* (for AMK and KAN), *eis* (for KAN), *gyrA* (for OFX and MXF), *rplC* and *23S* rRNA (for LZD); 9 primer sets for high resolution melt (HRM) analysis and two specific probe of the *pncA* gene (for PZA); and 1 primer and probe set for *M*. *tuberculosis 16S* rRNA gene as internal control; primers and probes sequences were shown in Table 1. The TAC format included 8 ports per card each port was compartmentalized into 48 reactions (Fig 1). Twenty microliters of input DNA was mixed with 50 μl of 2x MeltDoctor^™^ (SYTO^®^9) HRM master mix, 2 μl of 2.5 μM ROX reference dye and 28 μl of water to a 100 μl final volume. This was loaded into each port of the card, whereby each card included six clinical samples, *M*. *tuberculosis* H37Rv as wild-type control, and the positive control plasmid (Genewiz Inc., South Plainfield, NJ, USA) that contained primers and probes of all targets as a mutant control. The card was centrifuged twice at 1,200 rpm for 1 min, sealed, the loading ports were excised, and the card was run on a ViiA7 real-time PCR cycler (Life Technologies Corp., Carlsbad, CA, USA) for 2 hours following the cycling condition as described previously [6].

**Table 1:** Primers and probes used in the TB TAC v2.

Gene	Primers/Probes	Sequences	Assay region	Product size bp	References
*inhA*	Forward	5’-GCTCGTGGACATACCGATTT-3’	(-54)–codon 22	120	[6]
	Reverse	5’-TCCGGTAACCAGGACTGAAC-3’			[6]
	T(-8)C	VIC-5’-CGAGACGATAGGCTG-3’-MGB			[6]
	C(-15)T	VIC-5’-ACCTATCATCTCGCC-3’-MGB			[6]
	-8-15wt	VIC-5'-ACAACCTATCGTCTCGC-3'-MGB			This study
*katG*	Forward	5’-CTCGTATGGCACCGGAAC-3’	Codon 303–338	108	[6]
	Reverse	5’-CCGTACAGGATCTCGAGGAA-3’			[6]
	315 Thr^a^	HEX-5’-ATCA+CCA+CCG+GCA+TCG-3’			[12]
	315wt	VIC-5’-CGATGCCGCTGGT-3’-MGB			This study
*rpoB* Amplicon 1	Forward	5’-GCCGCGATCAAGGAGTTCT-3’	Codon 500–543	130	[6]
	Reverse	5’-CACGCTCACGTGACAGACC-3’			[6]
	511Pro	VIC-5’-AGCCAGCCGAGCC-3’-MGB			[6]
	513Leu	VIC-5’- CCAGCTGAGCCTAT-3’-MGB			[6]
	513Glu	VIC-5’-CTGAGCGAATTCA-3’-MGB			[6]
	516Val^a^	HEX-5’-AATTCA+TGG+TCC+AGA+ACAA-3’			[12]
	511wt	VIC-5’-CAGCCAGCTGAGCC-3’-MGB			This study
	513wt	VIC-5’-CTGAGCCAATTCAT-3’-MGB			This study
	513Lys	NED-5’-CTGAGCAAATTCA-3’-MGB			This study
	516wt^a^	HEX-5’-AATTCA+TGG+ACC+AGA+ACAA-3’			This study
	516Tyr	VIC-5’-CAATTCATGTACCAGAACA-3’-MGB			This study
*rpoB* Amplicon 2	Forward	5’-AGCCAGCTGAGCCAATTCAT-3’	Codon 509–543	103	[6]
	Reverse	5’-CACGCTCACGTGACAGACC-3’			[6]
	526Tyr	VIC-5’-CGGGGTTGACCTACA-3’-MGB			[6]
	526Asp	VIC-5’-GTTGACCGACAAGC-3’-MGB			[6]
	526Leu^a^	HEX-5’-GT+TGA+CCC+TCA+AGC-3’			[12]
	531Leu	VIC-5’- CCGACTGTTGGCGC-3’-MGB			[6]
	531Trp	NED-5’-CCGACTGTGGGCG-3’-MGB			[6]
	533Pro	VIC-5’- ACTGTCGGCGCCGG-3’-MGB			[6]
	526wt	VIC-5’-TTGACCCACAAGCG-3’-MGB			This study
	531wt	VIC-5’-CGACTGTCGGCGCT-3’-MGB			This study
	533wt	VIC-5’-TGTCGGCGCTGGG-3’-MGB			This study
*rrs* Amplicon 1	Forward	5’-AAGTCGGAGTCGCTAGTAATCG-3’	nt.1320-1427	104	[6]
	Reverse	5’-TTCGGGTGTTACCGACTTTC-3’			[6]
	A(1401)G	VIC-5’-CCCGTCGCGTCAT-3’-MGB			[13]
	1401wt	VIC-5’-GCCCGTCACGTCAT-3’-MGB			This study
*rrs* Amplicon 2	Forward	5’-AAAGTCGGTAACACCCGAAG-3’	nt.1409-1510	102	[6]
	Reverse	5’-CCGGTACGGCTACCTTGTTA-3’			[6]
	G(1484)T	VIC-5’-CGATTGGGACTAAGTC-3’-MGB			[6]
	1484wt	NED-5’-ATTGGGACGAAGTCGT-3’-MGB			This study
*eis*	Forward	5’-TGATCCTTTGCCAGACACTG-3’	(-73)—codon 10	103	[6]
	Reverse	5’-CTCGGTCGGGCTACACAG-3’			[6]
	C(-14)T	VIC-5’- CGGCATATGCTACAGT-3’-MGB			[6]
	G(-10)A	NED-5’-TATGCCACAATCGGATT-3’-MGB			[6]
	-10-15wt	VIC-5'-ATATGCCACAGTCGGATT-3'-MGB			This study
	C(-12)T	VIC-5’- CATATGCCATAGTCGGATT-3’-MGB			This study
*gyrA*	Forward	5’-CCGGTCGGTTGCCGAGACC-3’	Codon 75–109	107	[14]
	Reverse	5’-CCAGCGGGTAGCGCAGCGACCAG-3’			[14]
	90Val	VIC-5’-AGATCGACACGTCGCC-3’-MGB			[6]
	94Gly^b^	VIC-5’-CCAGGSTGCCGTAGATC-3’–MGB			[6]
	94Tyr^b^	VIC-5’-TCGATCTACTACASCCT-3’-MGB			[6]
	94Ala^b^	VIC-5’-ATCTACGCCASCCTG-3’-MGB			[6]
	90wt	VIC-5’-ATCGACGCGTCGCC-3’-MGB			This study
	94wt	VIC-5’-GATCTACGACAGCCTGG-3’-MGB			This study
	94Asn^b^	VIC-5’-TCGATCTACAACASCCTG-3’-MGB			This study
*rplC*	Forward	5’-TCCAAGGGCAAAGGTTTCG-3’	Codon 118–166	146	This study
	Reverse	5’-ATCCGGGTGCCCTTGAAC-3’			This study
	154wt	NED-5’-CGTGGCACATCCG-3’-MGB			This study
	154Arg	VIC-5’-TGGCACGTCCGCCGA-3’-MGB			This study
*23S* Amplicon 1	Forward	5’-CAAGTCAAGCAGGGACGAAA-3’	nt. 2359–2487	129	This study
	Reverse	5’-TCCCGTCGATATGGACTCTTG-3’			This study
	2447wt^c^	NED-5’-TACCCCGGGGATAACA-3’-MGB			This study
	G(2447)T^c^	VIC-5’-GTACCCCGGGTATAACA-3’-MGB			This study
*23S* Amplicon 2	Forward	5’-ACCTCGATGTCGGCTCGT-3’	nt. 2497–2617	124	This study
	Reverse	5’-GGCGGATAGAGACCGAACTG-3’			This study
	2576wt^c^	NED-5’-CCAGCTCGCGTGCC-3’-MGB			This study
	G(2576)T^c^	VIC-5’-ACCCAGCTAGCGTGC-3’-MGB			This study
*pncA* Amplicon 1	Forward	5’-GCGTCGGTAGGCAAACTG-3’	(-50)—codon 18	103	[6]
	Reverse	5’-GAGCCACCCTCGCAGAAGT-3’			[6]
*pncA* Amplicon 2	Forward	5’-ATCATCGTCGACGTGCAGA-3’	Codon 5–45	124	[6]
	Reverse	5’-CCACGACGTGATGGTAGTCC-3’			[6]
*pncA* Amplicon 3	Forward	5’-CGCCATCAGCGACTACCT-3’	Codon 30–66	113	[6]
	Reverse	5’-ACGAGGAATAGTCCGGTGTG-3’			[6]
	57Asp	VIC-5’-CCGGGTGACGACT-3’-MGB			[6]
*pncA* Amplicon 4	Forward	5’-ACCCGGGTGACCACTTCT-3’	Codon 53–86	102	[6]
	Reverse	5’-TGTCCAGACTGGGATGGAA-3’			[6]
	65Ser	VIC-5’-CGGACTATTCTTCGTCGT-3’-MGB			[6]
*pncA* Amplicon 5	Forward	5’-CATTGCGTCAGCGGTACTC-3’	Codon 71–113	128	[6]
	Reverse	5’-CCGTTCTCGTCGACTCCTT-3’			[6]
*pncA* Amplicon 6	Forward	5’-AATCGAGGCGGTGTTCTACA-3’	Codon 90–133	132	[6]
	Reverse	5’-ATACCGACCACATCGACCTC-3’			[6]
*pncA* Amplicon 7	Forward	5’-CACGCCACTGCTGAATTG-3’	Codon 114–149	109	[6]
	Reverse	5’-ATTGCGTACCGCGTCCTC-3’			[6]
*pncA* Amplicon 8	Forward	5’-AGGTCGATGTGGTCGGTATT-3’	Codon 128–162	107	[6]
	Reverse	5’-ACCCGCTGTCAGGTCCAC-3’			[6]
*pncA* Amplicon 9	Forward	5’-GAGGACGCGGTACGCAAT-3’	Codon 144–187	133	[6]
	Reverse	5’-ATCAGGAGCTGCAAACCAAC-3’			[6]
*16S*	Forward	5’-GGATAAGCCTGGGAAACTGGGT-3’	nt.142–259	118	[6] Modified
	Reverse	5’-ACCCCACCAACAAGCTGATAGG-3’			[6] Modified
	*M*.*tb*	VIC-5’-ACCACGGGATGCATGT-3’-MGB			[6] Modified

^a^ Locked nucleic acid bases are indicated by “+”,

^b^ a polymorphism at codon 95 Ser/Thr was included by using “S” G/C,

^c^ *E*. *coli* position number, underlined letters indicate the mutation position

### Sanger sequencing

All discrepant TAC results between sputum and cultured isolate, as well as between genotypic and phenotypic results were sequenced. The following seven loci were amplified by PCR: *inhA* and *katG* (INH), *rpoB* (RIF), *rrs* and *eis* (AMK,KAN), *gyrA* (OFX, MXF), and *pncA* (PZA) using the locus-specific primer of Campbell *et al* [15]. The *23S* rRNA (LZD) was amplified by primers designed in this study *23S*-F; 5’-CAGGTGGCGAGTGTAAATGC-3’ and *23S*-R; 5’-AGCGGTTATCCTGACCGAAC-3’. Each 25 μl PCR mixture contained 12.5 μl HotStarTaq master mix (Qiagen, Valencia, CA, USA), 0.25 μl of the forward and reverse 50 μM primers, 7 μl nuclease free water, and 5 μl of genomic DNA. PCR was performed on an CFX96 (Bio-Rad, Hercules, CA, USA) included an initial denaturation step at 95°C for 15 min, followed by 40 cycles of denaturation at 95°C for 30 sec, annealing at 60°C for 30 sec, and extension at 72°C for 30 sec, with a final extension step at 72°C for 10 min. PCR products were analyzed on 2% agarose-gels, verified PCR products were purified using MinElute^®^ 96 UF PCR Purification Kit (Qiagen, Valencia, CA, USA) followed the manufacturer’s protocol. Purified PCR products were measured spectrophotometrically, diluted with nuclease free water and mixed with primers then submitted to GeneWiz (Genewiz Inc., South Plainfield, NJ, USA) for DNA sequencing.

### Statistical analysis

Means or median were compared using *t* test or Mann-Whitney test, Receiver-operating characteristic (ROC) analysis was performed with PASW Statistics Software to define an optimal cycle threshold (Ct) cut-off. Data shown as mean ± standard deviations unless otherwise stated.

## Results

### The TAC version 2 assay

All new assays used in this study were specificity tested against wild-type *M*. *tuberculosis* H37Rv, previously characterized mutant isolates, and synthetic mutant control plasmids. This revealed no cross-reactivity, with only H37Rv amplifying with the wild-type assays and only the appropriate mutant amplifying with the mutant assays (S1 Fig). The final TAC version 2 layout is shown in Fig 1.

### Amplification results (SYTO9)

**Fig 2:**
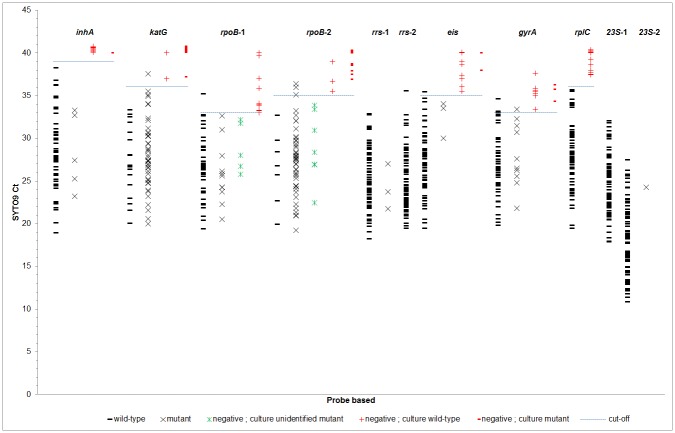
Amplification Ct cut-off. For each of 71 sputum samples the average SYTO9 Ct value for each of the 11 amplicons is shown. Average Ct are organized according to whether the sputum probe result was wild-type (black -), mutant (black x), or negative. Sputum probe negative results are further stratified into whether the cultured isolate was found to be wild-type (red +), mutant (red -), or to possess an “other” mutation (green X). ROC analysis was performed to examine the optimal Ct whereby SYTO9 to interpret the probe results.

This TAC version 2 was evaluated on 71 paired cultured isolates and their stored sputum sediment. All TAC testing was performed on site in Bangladesh and Thailand. We first examined the TAC sputum results. Each well contained an amplification dye (MeltDoctor SYTO9). The TAC generated 11 resistance-associated gene amplicons (*inhA*; *katG*; *eis*; *gyrA*; *rplC*; 2 amplicons for *rpoB*, *rrs*, and *23S*; not including 9 amplicons for *pncA* which are described below). We amplified for 40 cycles as per our original publication [6]. Each amplicon was replicated a different number of times based on the TAC configuration, for example twice for the *katG* amplicon (wells 4 and 5) and 8 times for *rpoB* amplicon 2 (wells 15–22). For each of the 11 amplicons, the SYTO9 Ct replicates were tight with an average coefficient of variation of 1% (S1 Table). Therefore, in Fig 2 we show the mean SYTO9 Ct for each of the 71 sputum’s 11 amplicons. These 781 Ct values are organized according to amplicon and whether the wild-type probe, a mutant probe, or neither was detected. This analysis revealed, in general, that the sputum amplicons were detected with SYTO9 Ct values in the 20–35 range. Late low-level detections, after Ct ~35, were reaching the limit of detection in sputum, where often the wild-type or mutant probes were negative (Fig 2). However if the amplicons were detected in the SYTO9 Ct 20–35 range without detection by wild-type or mutant probe, then typically the culture exhibited a rare mutation not interrogated by the TAC probes. Using these 71 sputum we were thus able to ascribe by ROC analysis the SYTO9 Ct cutoff for each amplicon below which one would expect (1) either the wild-type or mutant probe to be detected, or (2) if not detected then one could expect that the culture may reveal an “other” mutation; and above which one would conclude that the sputum was TB PCR negative or indeterminate. These Ct cutoffs ranged from 39 for *inhA* to 33 for *rpoB* amplicon 1, with an average of 35.

### Performance of TAC on direct specimens

Using these cutoffs we evaluated the performance of sputum TAC SYTO9 versus smear status. Among the 19 3+ smear positive specimens, TAC detected *inhA*, *katG*, *rpoB*, *eis*, *gyrA*, *rplC*, *and pncA* in all 19 specimens (Table 2). The detection rate for these genes fell to 94% for 2+ smear positive specimens, 76% for 1+ smear positive specimens, and 33% for smear negative specimens. We excluded SYTO9 results for *rrs* and 23S amplicons, because we noted that all specimens revealed detection regardless of smear status and upon sequencing some amplicons derived from other bacteria (e.g., *Corynebacterium*). Note that these primers work well on pure isolates as previously described [6, 7].

**Table 2:** Sensitivity of TAC to detect drug resistance on sputum samples.

AFB smear	Detectable samples														
	*inhA*		*katG*		*rpoB*		*eis*		*gyrA*		*rplC*		*pncA*		All 7 targets
	N	Ct (Ẋ ± SD)	N	Ct (Ẋ ± SD)	N	Ct (Ẋ ± SD)	N	Ct (Ẋ ± SD)	N	Ct (Ẋ ± SD)	N	Ct (Ẋ ± SD)	N	Ct (Ẋ ± SD)	N (%)
Positive 3+ (n = 19)	19	28.4 ± 3.9	19	27.7 ± 3.3	19	26.8 ± 2.3	19	25.8 ± 3.1	19	26.6 ± 2.7	19	27.8 ± 3.2	19	27.6 ± 3.1	19 (100)
Positive 2+ (n = 16)	15	25.7 ± 4.1	15	25.4 ± 3.7	15	24.9 ± 3.9	15	25.6 ± 3.9	16	25.6 ± 4.3	15	26.3 ± 4.2	15	26.0 ± 4.2	15 (94)
Positive 1+ (n = 21)	19	28.5 ± 4.4	17	28.0 ± 3.8	18	28.0 ± 3.9	18	28.3 ± 3.7	17	27.1 ± 3.5	18	29.0 ± 3.8	18	28.2 ± 3.8	16 (76)
Total positive (n = 56)	53	27.7 ± 4.2	51	27.1 ± 3.7	52	26.7 ± 3.6	52	26.6 ± 3.7	52	26.5 ± 3.5	52	27.8 ± 3.8	52	27.4 ± 3.8	50 (89)
Negative (n = 15)	9	32.3 ± 4.6	9	32.2 ± 3.9	8	31.0 ± 3.5	8	30.6 ± 3.6	8	30.7 ± 3.0	8	32.1 ± 3.4	7	31.1 ± 3.6	5 (33)
Total (n = 71)	62		60		60		60		60		60		59		55 (77)

Ct; cycle threshold, Ẋ; mean, SD; standard deviation

We then evaluated the accuracy of the wild-type or mutant probe results, which were labeled with a distinct fluorophore (VIC, HEX, or NED; see Table 1) from SYTO9. The probe result typically gave slightly later Ct than SYTO9 (average 2.4 ± 1.9; S2 Fig). The accuracy of the TAC probe result from sputum versus the paired isolate was evaluated (Table 3). Overall the sensitivity of the mutant probe on sputum was 98 ± 3% versus the mutant probe on the isolate as the standard. The specificity of the wild-type probe was 92 ± 14%. We sequenced the sputum and isolate for all discrepancies. Of the two false negative (wild-type sputum/mutant isolate) *katG* results, one was a heteroresistant isolate. The two false negative *rpoB* results on sputum were indeed false negatives (culture isolates were uninterrogated 526 mutant; 526Arg and 526Asn confirmed by sequencing), as were the 1 false positive mutant *rpoB*, *eis*, and *gyrA* mutant results (negative on both wild-type and mutant probe with the SYTO9 Ct near the cut-off). Additionally, we observed that of the 54 isolates that were *rpoB* mutant, 7 of them had mutations uninterrogated by TAC. Sequencing confirmed these were “other” mutations including 512Thr (n = 1), 526Cys (n = 1), 513Pro (n = 1), 526Asn (n = 2), and 526Arg (n = 2). The latter 3 are rare resistance-associated mutations [10] the former 2 are not resistance mutations. Additionally, 4 of the 54 *rpoB* mutant isolates were double mutant 511Pro + 526Gln (n = 1), 513Arg + 533Pro (n = 2), and 516Val + 531Leu (n = 1). For *rrs*, the mutant 1401 probe was 100% accurate, however the wild-type probe was non-specific since *rrs* primers were non-specific as mentioned above thus those results were excluded. For *pncA*, because of the long sequence requiring interrogation, we used high resolution melt analysis of 9 overlapping amplicons to obtain a wild-type versus non wild-type result. The sputum provided excellent sensitivity for detection of *pncA* mutants in the isolate (94%), however the specificity was only 64%, with 10 false positive mutant detections. Sequencing of discrepancies revealed that 8 of these were indeed wild-type but 2 were in fact correctly called by TAC as mutant.

**Table 3:** Performance of TAC to detect drug resistance on paired sputum and isolate.

Gene	Sputum	Culture		% Sensitivity (95% CI)	% Specificity (95% CI)	% Accuracy
		Mutant	Wild-type			
*inhA* (n = 62)	Mutant	5	0	100 (48, 100)	100 (94, 100)	100
	Wild-type	0	57			
*katG* (n = 60)	Mutant	43	0	96 (85, 99.5)	100 (78, 100)	97
	Wild-type	2	15			
*rpoB* (n = 60)	Mutant	52^a^	1	96 (87, 99.5)	83 (36, 100)	95
	Wild-type	2^b^	5			
*eis* (n = 60)	Mutant	2	1	100 (16, 100)	98 (91, 100)	98
	Wild-type	0	57			
*gyrA* (n = 60)	Mutant	9	1	100 (66, 100)	98 (90, 100)	98
	Wild-type	0	50			
*rplC* (n = 60)	Mutant	0	0	NA	100 (94, 100)	100
	Wild-type	0	60			
*pncA* (n = 59)	Mutant	29	10	94 (79, 99)	64 (44, 81)	80
	Wild-type	2	18			
Average ± SD				98 ± 3	92 ± 14	95 ± 7

^a^ 5 of these 52 had an “other” mutation (no detection with mutant or wild-type probe) that was seen in both sputum and culture and was confirmed by Sanger sequencing.

^b^ both cultured isolates had an uninterrogated mutation

NA; not applicable

### Correlation between genotypic and phenotypic DST

The TAC results from sputum and isolates were then compared to the phenotypic susceptibility results of the isolates as the gold-standard. The overall accuracy of the sputum TAC was 85% whereas the accuracy of the isolate TAC was 88% (P = 0.79, Table 4). This 88% genotypic-phenotypic accuracy is similar to what we have noted previously [6, 7] and genotypic results were generally supported by Sanger sequencing. For example, 2 samples were genotypically “false resistant” (mutant) in both sputum and isolate but were phenotypically susceptible. This included 1 each of RIF (*rpoB* 511Pro) and OFX/MXF (*gyrA* 94Gly) (Table 5). Likewise, there were 8 genotypically “false susceptible” (wild-type) isolates that were phenotypically resistant, 4 for INH (*inhA*/*katG*), 4 for OFX/MXF (*gyrA*), where sequencing confirmed all to be wild-type (Table 5). We do not show the injectable results in Table 4 because of the above-mentioned nonspecificity of the *rrs* primers and wild-type probe, however we found 95% accuracy versus phenotypic DST using the *eis* wild-type and mutant result in conjunction with the *rrs* 1401 mutant result on sputum. The accuracy between genotypic HRM and phenotypic susceptibility testing for PZA was 68% for both sputum and isolates and reflected both HRM-Sanger and Sanger-phenotypic discordance. We could not adequately evaluate LZD results because there was only 1 phenotypically resistant isolate (which was *rplC*/*23S* wild-type) and only 1 genotypically resistant (*23S* mutant G2576T) sputum/isolate that was phenotypically susceptible, the rest of the results were all TAC sputum and isolate *rplC*/*23S* wild-type and phenotypically susceptible.

**Table 4:** Correlation between genotypic and phenotypic drug susceptibility test.

Drug	Gene	TAC Sputum	Phenotypic DST		%Sensitivity (95% CI)	%Specificity (95% CI)	%Accuracy	TAC Culture	Phenotypic DST		%Sensitivity (95% CI)	%Specificity (95% CI)	%Accuracy
			R	S					R	S			
INH	*inhA/katG* (n = 59)	Mt	45	0	88 (76.1, 95.6)	100 (63.1, 100)	90	Mt	47	0	92 (81.1, 97.8)	100 (63.1, 100)	93
		Wt	6	8				Wt	4	8			
RIF	*rpoB* (n = 60)	Mt	51	2	96 (87.0, 99.5)	71 (29.0, 96.3)	93	Mt	53	1	100 (93.3, 100)	86 (42.1, 99.6)	98
		Wt	2	5				Wt	0	6			
OFX/MXF	*gyrA* (n = 60)	Mt	8	2	67 (34.9, 90.1)	96 (85.7, 99.5)	90	Mt	8	1	67 (34.9, 90.1)	98 (88.9, 99.9)	92
		Wt	4	46				Wt	4	47			
PZA	*pncA* (n = 59)	Mt	17	16	85 (62.1, 96.8)	59 (42.1, 74.4)	68	Mt	14	13	70 (45.7, 88.1)	67 (49.8, 80.9)	68
		Wt	3	23				Wt	6	26			
Average ± SD					84 ± 12	82 ± 20	85 ± 12				82 ± 16	88 ± 15	88 ± 13

Mt; mutant, Wt; wild-type, R; resistance S; susceptible, Ẋ; mean, SD; standard deviation

**Table 5:** Sequencing confirmation of the discordance samples between TAC and phenotypic DST.

Gene/Drug	Sputum				Culture			
	TAC	Sanger Sequencing	Phenotypic DST	No. of samples with discrepancy	TAC	Sanger Sequencing	Phenotypic DST	No. of samples with discrepancy
*inhA*,*katG*/INH	Wild-type	Wild-type	Resistant	6	Wild-type	Wild-type	Resistant	4
*rpoB*/RIF	Wild-type	526His+526Arg	Resistant	1				
	Wild-type	His526Asn	Resistant	1				
	511Pro	Leu511Pro	Susceptible	1	511Pro	Leu511Pro	Susceptible	1
	“other mutation”	Wild-type	Susceptible	1				
*gyrA*/OFX,MXF	Wild-type	Wild-type	Resistant	4	Wild-type	Wild-type	Resistant	4
	94Gly	Asp94Gly	Susceptible	1^a^	94Gly	Asp94Gly	Susceptible	1^a^
	“other mutation”	Wild-type	Susceptible	1				
*pncA*/PZA	Wild-type	Wild-type	Resistant	1	Wild-type	Wild-type	Resistant	3
	Wild-type	N/A	Resistant	1	Wild-type	Pro54Leu	Resistant	1
	65Ser^b^	Ser65Ser^b^ + Ins 392 GG	Resistant	1	65Ser^b^	Ser65Ser^b^ + Ins 392 GG	Resistant	1
					Wild-type	Pro62Leu	Resistant	1
	Mutant	Wild-type	Susceptible	12	Mutant	Wild-type	Susceptible	8
	Mutant	Ile31Thr	Susceptible	1	Mutant	Ile31Thr	Susceptible	1
	Mutant	Ala38Val	Susceptible	1	Mutant	Ala38Val	Susceptible	1
	65Ser^b^ + Mutant	Ser65Ser^b^	Susceptible	1	65Ser^b^ + Mutant	Ser65Ser^b^	Susceptible	3
	Mutant	N/A	Susceptible	1				

^a^Moxifloxacin susceptible, ofloxacin not done

^b^Silent mutation (TCG-TCC)

N/A means we did not perform sequencing or sequencing failed

## Discussion

In the first study [6] we developed a TAC version that included 27 primer pairs and 40 probes to interrogate critical regions of the *inhA*, *katG*, *rpoB*, *embB*, *rpsL*, *rrs*, *eis*, *gyrA*, *gyrB*, and *pncA* genes. TAC yielded 96.1% accuracy compared to Sanger sequencing and 87% concordance with culture based susceptibility testing. The TAC was then field tested in Bangladesh, Tanzania, and Thailand and again yielded 87% accuracy versus traditional culture based DST, or 94% accuracy if a consensus gold standard was used that included MIC results [7]. These two studies were evaluated on cultured isolates and provided an early DST result several weeks sooner than phenotypic DST, however still required waiting for cultured growth. To speed turnaround time here we evaluated the TAC method directly on sputum. We found that it provided a highly predictive genotypic (average 95% accuracy for 7 genes) and phenotypic (average 85% accuracy for 5 drugs) susceptibility result. The assay worked well on smear positive sputum (89% of which yielded an interpretable result), excellently on highly smear positive sputum (94–100%), but significantly worse on smear negative sputum (33%, P < 0.05).

This sensitivity on smear positive sputum and poor yield on smear negative sputum was not surprising given the well-known difficulties of amplifying TB DNA from direct specimens. Even an excellent commercial method such as GeneXpert, which utilizes heminested PCR and only amplifies one gene, only succeeds in 72% of smear negative specimens under clinical trial conditions [16]. Our study was performed under field conditions, the sediments were stored at -80°C for at least 6 weeks prior to testing, and the TAC method itself amplifies in small 1μl volumes which limits input DNA. In future work we will evaluate extraction methods to increase the volume of input DNA. Thus while at present one would not advocate TAC DST as reliable on smear negative sputum, its performance on smear positive material is robust. For instance, the sensitivity of TAC on sputum to accurately predict *rpoB* mutations and RIF resistance on the subsequent isolate was 96%, in line with that of GeneXpert or MTBDR*plus* [16, 17].

A benefit of TAC is the ability to interrogate and identify specific mutations—here we identified 30 unique SNPs. Given the spread of databases of TB mutations in diverse genes, some of which have high likelihood ratios for resistance, others moderate, others low, others no likelihood at all ([18]; ReseqTB.org) the future of molecular diagnostics for TB is quickly outgrowing probe based results that yield a crude “wild-type” or “some mutation” output without sequence identity. For instance, even though we included the 12 most prevalent resistance-associated *rpoB* mutations, we still encountered 7 of 71 (10%) MDR TB specimens that had “other” *rpoB* mutations—some of which were rare resistance-associated mutations others not resistance-associated at all. This shows the value of including wild-type probes—if we found strong amplification with SYTO9 but no detection with either the wild-type or mutant probe then we knew an “other” mutation was present. Four of these seven were tested by GeneXpert RIF, which misdiagnosed half of them (versus phenotypic DST, data not shown). In the United States 21% of GeneXpert RIF R specimens have silent mutations or no mutation at all, and another 14% have disputed mutations [19]. Thus the inadequacy of relying on probe mismatch without sequence identity is increasingly clear. The TAC format is modular, thus our next version includes every vetted *rpoB* resistance-associated mutation, including the 513Pro, 526Asn, and 526Arg mutations noted here. It is also informative to know the specific mutation since some are associated with low-level resistance, where potentially rifampin at higher dose or rifabutin could be used [20].

Most importantly, TAC is easy to perform in the field. Our laboratories in Bangladesh and Thailand also use line probe assays, but these methods are time-consuming and laborious. Sanger sequencing is generally not available locally and requires international shipments. TAC cards, once procured, are stable at ambient temperature and lab preparation time involves only DNA extraction and loading the card. We have used this platform extensively on thousands of stool specimens around the world and have documented excellent reproducibility [21].

There were limitations to this work. A lesion of the current TAC is the *pncA* result. *pncA* mutations are scattered throughout the 561 base pair gene and an upstream promoter-containing region, thus genotypic assay design is difficult. We used an HRM method of 9 overlapping amplicons that discerns wild-type or variant to the H37Rv control. We included probes for the *M*. *bovis* specific mutation probe and the common 65Ser silent mutation. But the performance of this assay on isolates has hovered around 80–90% accuracy versus Sanger sequencing, and was unchanged using direct sputum. Following the logic of the *rpoB* discussion above, given the large number of non-resistance associated *pncA* mutations, our next TAC version will include individual probes for all of the accepted resistance mutations. This will require an additional port dedicated to *pncA*.

Another limitation is that we did not evaluate the assay on large numbers of drug susceptible and TB negative sputa, so we do not have complete specificity data from these contexts. That said, the majority of the isolates tested here were susceptible to second line drugs, so indeed our specificity confidence intervals were tight. Another limitation was that specificity was a problem with the *rrs and 23S* genes due to non-TB specificity of the primers. This is not a problem when using isolates but is a problem in the contaminated setting of sputum and hinders performance of this current TAC to detect XDR-TB. Better design of the wild-type *rrs* and *23S* assays is underway. Another comment is that our laboratory used 2.0 μg/mL for the critical concentration of ofloxacin in LJ not the recently updated 4.0 μg/mL, which may have undercalled resistance. Finally, we included linezolid resistance associated mutations based on the existing literature, most of which is in vitro [22], but the prevalence of such mutations, their specificity [23, 24], and the prevalence of linezolid resistance, is currently low so the utility of these assays remains to be seen.

TAC on smear positive sputum could potentially be used alongside other diagnostic and treatment algorithms. For example, in resource limited settings that use only GeneXpert and line probe assays, TAC with assays for *gyrA* and *pncA* could be used to license WHO-endorsed short course MDR treatment if *gyrA* and *pncA* had no resistance mutations; or TAC with a broader set of assays could be used guide individualized therapy.

## Supporting information

S1 Fig Specificity testing of the assays. Specificity testing of the new assays in this TAC version 2 was performed on the 384 well plate format. Assays were tested with wild-type (H37Rv) and the well-known mutant isolates including *inhA*-8C, -15T, *katG*315Thr, *rpoB*511Pro, 513Glu, 513Leu, 516Val, 516Tyr, 526Tyr, 526Asp, 526Leu, 531Leu, 531Trp, 533Pro, *rrs*1401G, 1484T, *eis*-14T, -10A, *gyrA*90Val, 94Gly, 94Tyr, 94Ala, 94Asn, synthetic plasmid controls included p*rpoB*513Lys, p*eis*-12T, p*rplC*154Arg, p*23S*2447T, and p*23S*2576T. Each assay shows amplification of only the appropriate wild-type or mutant.
(TIF)

S2 Fig Correlation between SYTO9 and probe results on 71 sputum for the 11 amplicons. Note average probe-based Ct is 2.4 ± 1.9 below the SYTO9 Ct of *inhA* (A), *katG* (B), *rpoB*-1 (C), *rpoB*-2 (D), *rrs*-1 (E), *rrs*-2 (F), *eis* (G), *gyrA* (H), *rplC* (I), *23S*-1 (J), and *23S*-2 (K). Each graph shows the probe result (wild-type, mutant, or negative). If negative, results are stratified whether culture was wild type, mutant, or had an uninterrogated mutation.
(TIF)

S1 Table Coefficient of variation of qPCR results (SYTO9 Ct) of 71 sputum samples. (DOCX)
