# Supplementary figures and images for: Performance of TaqMan array card to detect TB drug resistance on direct specimens
Source: PLoS One. 2017 May 4;12(5):e0177167. doi: 10.1371/journal.pone.0177167 (PMC5417650; doi:10.1371/journal.pone.0177167)

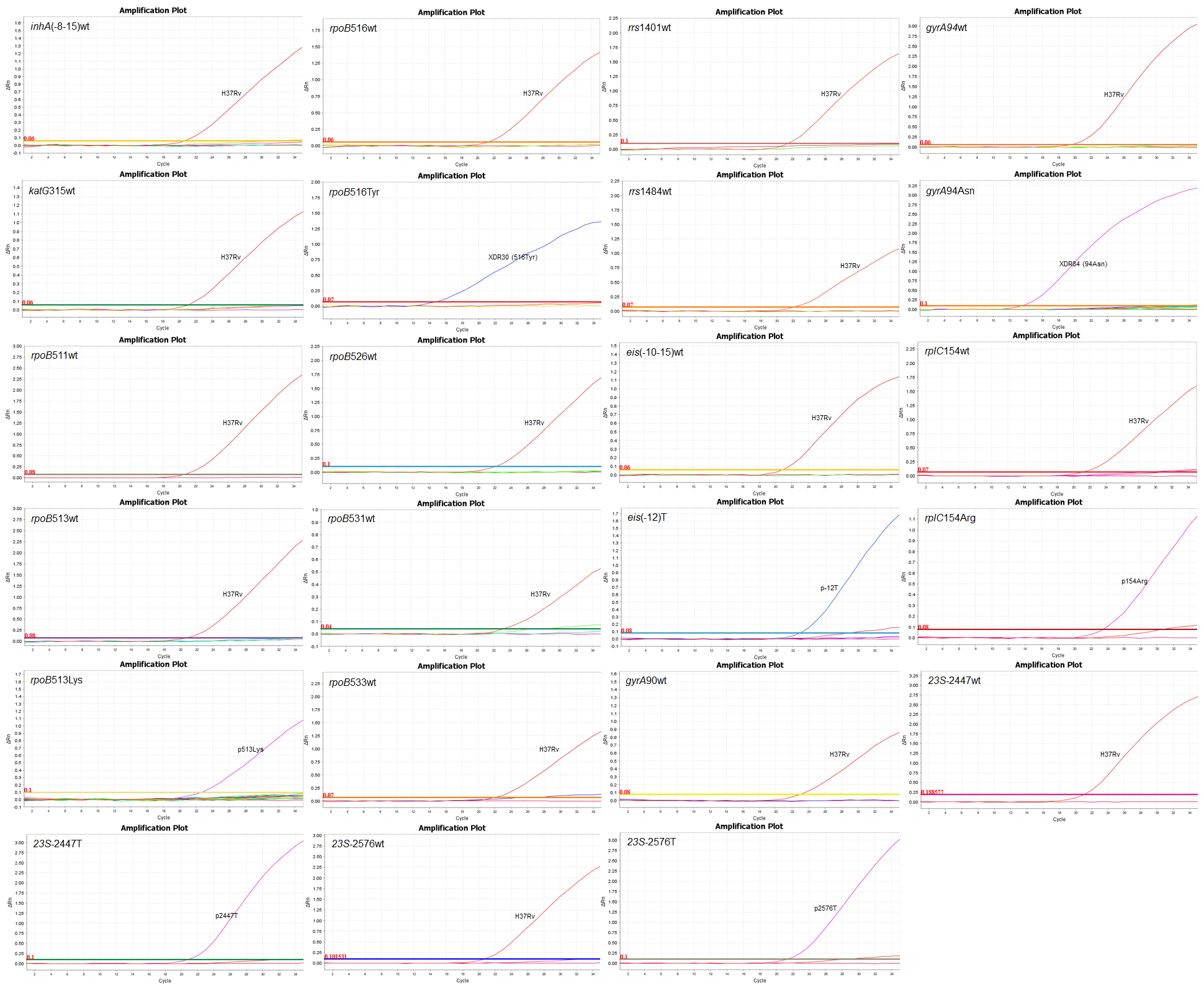

Supplement: S1 Fig — Specificity testing of the new assays in this TAC version 2 was performed on the 384 well plate format. Assays were tested with wild-type (H37Rv) and the well-known mutant isolates including inhA-8C, -15T, katG315Thr, rpoB511Pro, 513Glu, 513Leu, 516Val, 516Tyr, 526Tyr, 526Asp, 526Leu, 531Leu, 531Trp, 533Pro, rrs1401G, 1484T, eis-14T, -10A, gyrA90Val, 94Gly, 94Tyr, 94Ala, 94Asn, synthetic plasmid controls included prpoB513Lys, peis-12T, prplC154Arg, p23S2447T, and p23S2576T. Each assay shows amplification of only the appropriate wild-type or mutant. (TIF) [file pone.0177167.s001.tif]

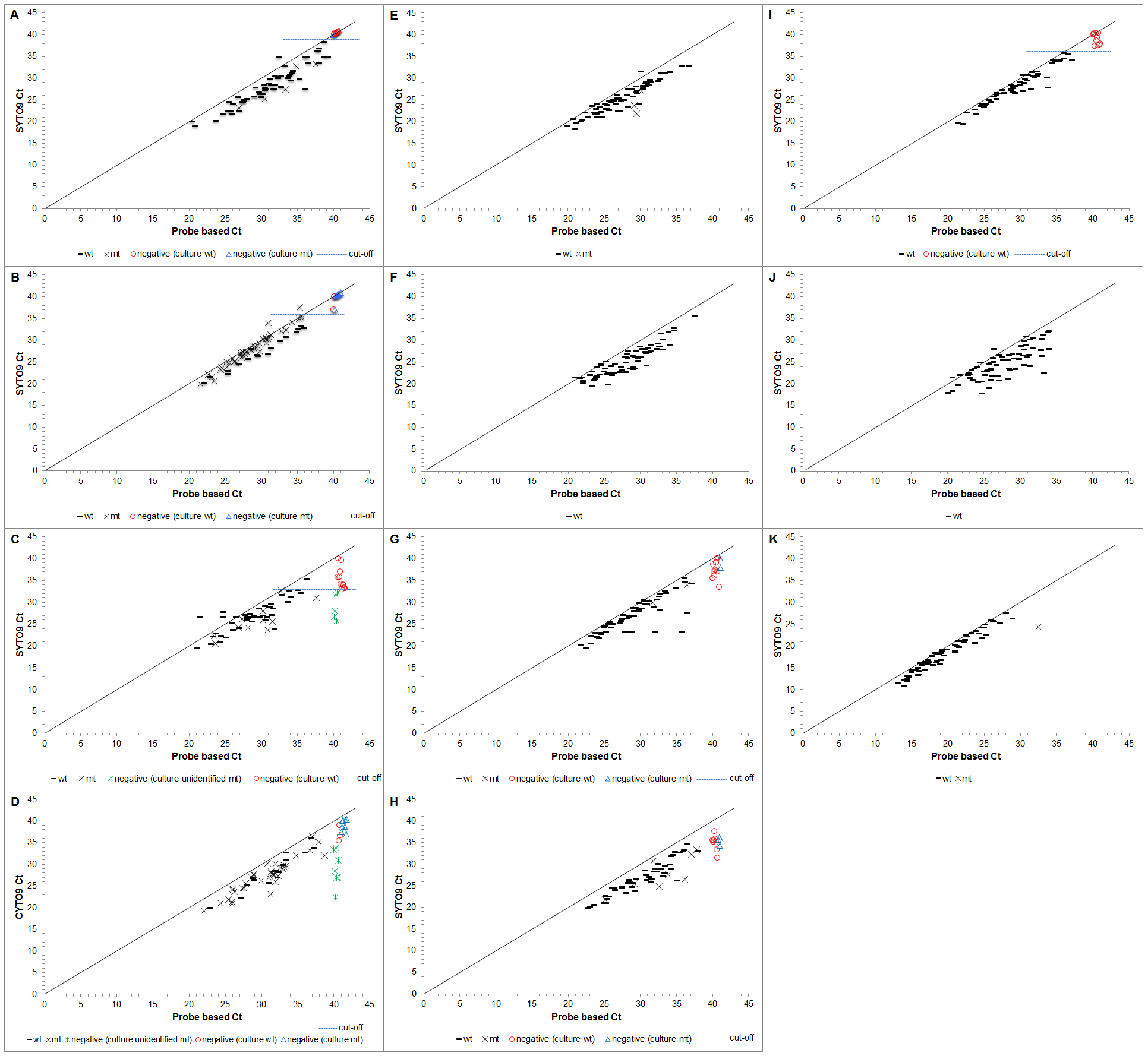

Supplement: S2 Fig — Note average probe-based Ct is 2.4 ± 1.9 below the SYTO9 Ct of inhA (A), katG (B), rpoB-1 (C), rpoB-2 (D), rrs-1 (E), rrs-2 (F), eis (G), gyrA (H), rplC (I), 23S-1 (J), and 23S-2 (K). Each graph shows the probe result (wild-type, mutant, or negative). If negative, results are stratified whether culture was wild type, mutant, or had an uninterrogated mutation. (TIF) [file pone.0177167.s002.tif]
