# Supplementary material for: Performance of TaqMan array card to detect TB drug resistance on direct specimens
Source: PLoS One. 2017 May 4;12(5):e0177167. doi: 10.1371/journal.pone.0177167 (PMC5417650; doi:10.1371/journal.pone.0177167)
Supplement: S1 Table — (DOCX) [file pone.0177167.s003.docx]

**S1 Table.** Coefficient of variation of qPCR results (SYTO9 Ct) of 71 sputum samples

| Sample ID | *inhA* (3 replicates) | | | *katG* (2 replicates) | | | *rpoB-*1 (8 replicates) | | | *rpoB-*2 (8 replicates) | | | *rrs-*1 (2 replicates) | | | *eis* (3 replicates) | | | *gyrA* (7 replicates) | | |
| --- | --- | --- | --- | --- | --- | --- | --- | --- | --- | --- | --- | --- | --- | --- | --- | --- | --- | --- | --- | --- | --- |
|  | Mean | SD | CV | Mean | SD | CV | Mean | SD | CV | Mean | SD | CV | Mean | SD | CV | Mean | SD | CV | Mean | SD | CV |
| XR0006 | 24.95 | 0.21 | 0.01 | 23.22 | 0.18 | 0.01 | 23.36 | 0.23 | 0.01 | 24.08 | 0.44 | 0.02 | 21.86 | 0.37 | 0.02 | 23.27 | 0.57 | 0.02 | 23.27 | 0.42 | 0.02 |
| XR0008 | 30.80 | 1.69 | 0.05 | 26.15 | 0.39 | 0.01 | 26.58 | 0.64 | 0.02 | 26.99 | 0.36 | 0.01 | 24.62 | 0.69 | 0.03 | 23.27 | 0.27 | 0.01 | 25.97 | 0.42 | 0.02 |
| XR0009 | 32.69 | 0.21 | 0.01 | 30.27 | 0.17 | 0.01 | 26.95 | 0.71 | 0.03 | 29.13 | 0.83 | 0.03 | 24.74 | 0.01 | 0.00 | 23.27 | 0.74 | 0.03 | 29.87 | 0.47 | 0.02 |
| XR0012 | 28.80 | 0.64 | 0.02 | 27.37 | 0.58 | 0.02 | 27.03 | 0.43 | 0.02 | 27.66 | 0.36 | 0.01 | 26.03 | 0.84 | 0.03 | 23.27 | 0.37 | 0.02 | 27.48 | 0.46 | 0.02 |
| XR0013 | 34.75 | 0.61 | 0.02 | 32.33 | 0.50 | 0.02 | 30.11 | 0.36 | 0.01 | 33.42 | 1.27 | 0.04 | 24.94 | 0.95 | 0.04 | 23.27 | 0.80 | 0.03 | 31.47 | 2.40 | 0.08 |
| XR0015 | 28.40 | 0.34 | 0.01 | 27.32 | 0.50 | 0.02 | 27.30 | 0.43 | 0.02 | 27.62 | 0.36 | 0.01 | 25.42 | 0.68 | 0.03 | 23.27 | 0.19 | 0.01 | 26.49 | 0.80 | 0.03 |
| XR0005 | 30.38 | 0.34 | 0.01 | 30.71 | 0.03 | 0.00 | 26.74 | 0.68 | 0.03 | 30.17 | 0.46 | 0.02 | 20.51 | 0.10 | 0.00 | 30.62 | 0.47 | 0.02 | 29.00 | 0.77 | 0.03 |
| XR0017 | 24.16 | 0.36 | 0.01 | 24.81 | 0.46 | 0.02 | 26.74 | 0.31 | 0.01 | 24.40 | 0.31 | 0.01 | 23.64 | 0.31 | 0.01 | 24.51 | 0.35 | 0.01 | 24.56 | 0.37 | 0.02 |
| XR0018 | 25.80 | 0.89 | 0.03 | 26.64 | 0.29 | 0.01 | 26.74 | 0.36 | 0.01 | 27.37 | 0.65 | 0.02 | 23.39 | 0.96 | 0.04 | 26.22 | 0.73 | 0.03 | 26.41 | 0.67 | 0.03 |
| XR0022 | 26.32 | 1.23 | 0.05 | 26.86 | 0.00 | 0.00 | 26.74 | 0.59 | 0.02 | 25.71 | 0.29 | 0.01 | 24.55 | 0.07 | 0.00 | 25.44 | 0.64 | 0.03 | 25.71 | 0.40 | 0.02 |
| XR0027 | 27.37 | 2.19 | 0.08 | 27.99 | 0.23 | 0.01 | 26.74 | 0.51 | 0.02 | 27.95 | 0.43 | 0.02 | 24.70 | 0.45 | 0.02 | 28.65 | 0.20 | 0.01 | 28.53 | 0.29 | 0.01 |
| XR0030 | 18.92 | 0.92 | 0.05 | 19.99 | 0.10 | 0.01 | 26.74 | 0.32 | 0.01 | 19.26 | 0.58 | 0.03 | 18.24 | 0.14 | 0.01 | 19.44 | 0.67 | 0.03 | 19.84 | 0.33 | 0.02 |
| XR0032 | 38.27 | 2.99 | 0.08 | 33.97 | 0.32 | 0.01 | 25.04 | 2.62 | 0.10 | 29.12 | 3.73 | 0.13 | 40.00 | 0.00 | 0.00 | 31.37 | 2.69 | 0.09 | 27.61 | 2.77 | 0.10 |
| XR0036 | 32.94 | 0.33 | 0.01 | 31.21 | 0.06 | 0.00 | 28.05 | 3.00 | 0.11 | 30.90 | 3.03 | 0.10 | 26.43 | 2.27 | 0.09 | 28.20 | 2.07 | 0.07 | 28.80 | 2.08 | 0.07 |
| XR0038 | 27.63 | 0.37 | 0.01 | 26.54 | 0.09 | 0.00 | 25.53 | 0.71 | 0.03 | 23.08 | 4.46 | 0.19 | 23.39 | 0.31 | 0.01 | 26.58 | 0.27 | 0.01 | 25.76 | 0.63 | 0.02 |
| XR0044 | 26.34 | 0.48 | 0.02 | 25.18 | 0.25 | 0.01 | 24.14 | 0.61 | 0.03 | 25.63 | 0.70 | 0.03 | 21.02 | 0.35 | 0.02 | 25.22 | 0.31 | 0.01 | 25.15 | 1.43 | 0.06 |
| XR0048 | 23.24 | 1.27 | 0.05 | 21.60 | 0.68 | 0.03 | 20.87 | 1.76 | 0.08 | 21.37 | 1.23 | 0.06 | 20.93 | 0.06 | 0.00 | 22.10 | 1.49 | 0.07 | 21.79 | 0.31 | 0.01 |
| XR0051 | 27.84 | 1.21 | 0.04 | 27.42 | 0.08 | 0.00 | 26.57 | 0.37 | 0.01 | 27.55 | 2.10 | 0.08 | 21.07 | 0.18 | 0.01 | 28.79 | 0.58 | 0.02 | 27.23 | 1.27 | 0.05 |
| XR0016 | 29.84 | 1.00 | 0.03 | 29.35 | 0.70 | 0.02 | 23.75 | 0.53 | 0.02 | 31.11 | 0.63 | 0.02 | 20.32 | 0.11 | 0.01 | 29.79 | 0.07 | 0.00 | 27.03 | 1.34 | 0.05 |
| XR0023 | 24.50 | 0.08 | 0.00 | 23.90 | 0.29 | 0.01 | 21.88 | 0.67 | 0.03 | 23.71 | 0.55 | 0.02 | 22.03 | 0.27 | 0.01 | 24.34 | 0.59 | 0.02 | 24.03 | 0.58 | 0.02 |
| XR0028 | 25.68 | 0.32 | 0.01 | 25.10 | 1.01 | 0.04 | 24.28 | 0.23 | 0.01 | 26.36 | 0.55 | 0.02 | 21.76 | 1.24 | 0.06 | 25.11 | 0.28 | 0.01 | 24.82 | 0.72 | 0.03 |
| XR0029 | 20.11 | 0.25 | 0.01 | 20.05 | 0.01 | 0.00 | 19.43 | 0.45 | 0.02 | 19.95 | 0.56 | 0.03 | 19.05 | 0.28 | 0.01 | 20.12 | 0.11 | 0.01 | 20.19 | 0.20 | 0.01 |
| XR0031 | 25.22 | 0.38 | 0.01 | 24.72 | 0.16 | 0.01 | 23.64 | 0.53 | 0.02 | 24.41 | 0.17 | 0.01 | 22.15 | 0.65 | 0.03 | 24.67 | 0.32 | 0.01 | 24.53 | 0.55 | 0.02 |
| XR0042 | 21.64 | 0.50 | 0.02 | 20.59 | 0.05 | 0.00 | 20.43 | 0.53 | 0.03 | 21.01 | 0.60 | 0.03 | 20.05 | 0.04 | 0.00 | 21.73 | 0.34 | 0.02 | 21.02 | 0.74 | 0.04 |
| XR0003 | 27.26 | 0.56 | 0.02 | 28.00 | 0.59 | 0.02 | 26.51 | 0.68 | 0.03 | 27.83 | 0.31 | 0.01 | 22.92 | 0.08 | 0.00 | 27.86 | 0.18 | 0.01 | 27.56 | 0.43 | 0.02 |
| XR0007 | 24.43 | 0.73 | 0.03 | 24.55 | 0.13 | 0.01 | 24.27 | 0.54 | 0.02 | 25.34 | 0.65 | 0.03 | 24.02 | 0.27 | 0.01 | 24.22 | 0.54 | 0.02 | 24.47 | 0.44 | 0.02 |
| XR0014 | 20.14 | 0.48 | 0.02 | 21.56 | 0.29 | 0.01 | 20.54 | 0.68 | 0.03 | 20.91 | 0.36 | 0.02 | 19.73 | 0.45 | 0.02 | 20.52 | 0.39 | 0.02 | 20.56 | 0.52 | 0.03 |
| XR0021 | 22.34 | 0.74 | 0.03 | 23.79 | 0.08 | 0.00 | 22.31 | 0.35 | 0.02 | 22.68 | 0.47 | 0.02 | 21.84 | 0.28 | 0.01 | 23.01 | 0.25 | 0.01 | 22.56 | 0.53 | 0.02 |
| XR0024 | 27.64 | 0.33 | 0.01 | 28.64 | 0.52 | 0.02 | 27.99 | 0.56 | 0.02 | 28.24 | 0.51 | 0.02 | 25.24 | 0.42 | 0.02 | 27.89 | 1.61 | 0.06 | 26.41 | 0.86 | 0.03 |
| XR0025 | 22.41 | 0.79 | 0.04 | 22.31 | 0.04 | 0.00 | 22.29 | 0.62 | 0.03 | 22.29 | 0.36 | 0.02 | 21.94 | 0.10 | 0.00 | 22.72 | 0.54 | 0.02 | 22.64 | 0.36 | 0.02 |
| XR0033 | 25.65 | 0.09 | 0.00 | 25.72 | 0.18 | 0.01 | 26.13 | 0.08 | 0.00 | 25.77 | 0.16 | 0.01 | 25.43 | 0.15 | 0.01 | 26.16 | 0.04 | 0.00 | 24.68 | 0.22 | 0.01 |
| XR0041 | 30.02 | 0.11 | 0.00 | 30.72 | 0.05 | 0.00 | 25.82 | 0.25 | 0.01 | 30.05 | 0.43 | 0.01 | 23.80 | 0.25 | 0.01 | 30.63 | 0.31 | 0.01 | 27.89 | 0.92 | 0.03 |
| XR0043 | 26.69 | 0.15 | 0.01 | 27.01 | 0.09 | 0.00 | 25.87 | 0.21 | 0.01 | 36.49 | 2.74 | 0.08 | 24.95 | 0.14 | 0.01 | 27.11 | 0.15 | 0.01 | 25.46 | 0.38 | 0.01 |
| XR0045 | 21.85 | 0.19 | 0.01 | 22.20 | 0.16 | 0.01 | 22.20 | 0.09 | 0.00 | 21.81 | 0.07 | 0.00 | 21.07 | 0.04 | 0.00 | 22.36 | 0.08 | 0.00 | 21.03 | 0.15 | 0.01 |
| XR0050 | 28.56 | 0.27 | 0.01 | 28.79 | 0.13 | 0.00 | 26.48 | 0.28 | 0.01 | 28.36 | 0.25 | 0.01 | 22.84 | 0.31 | 0.01 | 28.74 | 0.06 | 0.00 | 26.47 | 0.44 | 0.02 |
| XR0054 | 22.55 | 0.13 | 0.01 | 22.97 | 0.01 | 0.00 | 22.87 | 0.16 | 0.01 | 22.43 | 0.08 | 0.00 | 22.01 | 0.03 | 0.00 | 23.04 | 0.35 | 0.02 | 22.01 | 0.53 | 0.02 |
| XR0062 | 24.59 | 0.06 | 0.00 | 25.84 | 0.14 | 0.01 | 25.59 | 0.15 | 0.01 | 28.38 | 0.47 | 0.02 | 24.96 | 0.04 | 0.00 | 25.70 | 0.18 | 0.01 | 25.70 | 0.22 | 0.01 |
| XR0066 | 25.26 | 0.26 | 0.01 | 26.41 | 0.13 | 0.00 | 25.94 | 0.12 | 0.00 | 26.32 | 0.12 | 0.00 | 23.77 | 0.16 | 0.01 | 26.27 | 0.30 | 0.01 | 26.25 | 0.13 | 0.00 |
| MR0239 | 33.62 | 1.74 | 0.05 | 33.37 | 0.76 | 0.02 | 31.19 | 0.37 | 0.01 | 32.71 | 0.74 | 0.02 | 27.71 | 0.16 | 0.01 | 32.64 | 0.21 | 0.01 | 32.09 | 0.33 | 0.01 |
| MR0266 | 33.48 | 1.64 | 0.05 | 34.69 | 0.43 | 0.01 | 31.54 | 0.45 | 0.01 | 33.86 | 0.55 | 0.02 | 24.17 | 0.01 | 0.00 | 34.29 | 1.07 | 0.03 | 33.18 | 0.67 | 0.02 |
| MR0283 | 25.84 | 0.20 | 0.01 | 26.69 | 0.11 | 0.00 | 26.39 | 0.25 | 0.01 | 26.79 | 0.16 | 0.01 | 22.50 | 0.11 | 0.00 | 26.77 | 0.12 | 0.00 | 26.68 | 0.15 | 0.01 |
| XR0120 | 27.97 | 0.16 | 0.01 | 28.51 | 0.07 | 0.00 | 28.59 | 0.31 | 0.01 | 28.04 | 0.15 | 0.01 | 22.46 | 0.07 | 0.00 | 28.56 | 0.12 | 0.00 | 28.31 | 0.22 | 0.01 |
| XR0125 | 29.44 | 0.21 | 0.01 | 29.42 | 0.26 | 0.01 | 27.07 | 0.20 | 0.01 | 28.94 | 0.34 | 0.01 | 23.10 | 0.28 | 0.01 | 29.51 | 0.18 | 0.01 | 29.00 | 0.27 | 0.01 |
| XR0127 | 31.13 | 0.14 | 0.00 | 32.53 | 0.38 | 0.01 | 32.63 | 0.40 | 0.01 | 32.69 | 0.51 | 0.02 | 27.43 | 0.32 | 0.01 | 32.04 | 0.29 | 0.01 | 32.67 | 0.33 | 0.01 |
| XR0133 | 24.64 | 0.15 | 0.01 | 25.07 | 0.07 | 0.00 | 25.13 | 0.09 | 0.00 | 24.44 | 0.14 | 0.01 | 22.57 | 0.03 | 0.00 | 25.07 | 0.19 | 0.01 | 24.81 | 0.12 | 0.00 |
| MR0343 | 27.46 | 0.21 | 0.01 | 28.09 | 0.10 | 0.00 | 29.10 | 0.11 | 0.00 | 28.42 | 0.15 | 0.01 | 25.72 | 0.26 | 0.01 | 28.32 | 0.20 | 0.01 | 28.25 | 0.29 | 0.01 |
| MR0350 | 29.85 | 0.40 | 0.01 | 29.84 | 0.40 | 0.01 | 28.64 | 0.22 | 0.01 | 29.76 | 0.29 | 0.01 | 24.13 | 0.29 | 0.01 | 29.73 | 0.43 | 0.01 | 29.63 | 0.36 | 0.01 |
| SP5444 | 28.39 | 0.42 | 0.01 | 27.76 | 0.18 | 0.01 | 26.90 | 0.18 | 0.01 | 26.98 | 0.27 | 0.01 | 27.13 | 0.13 | 0.00 | 28.08 | 0.23 | 0.01 | 26.53 | 0.21 | 0.01 |
| SP0835 | 27.45 | 0.22 | 0.01 | 26.82 | 0.09 | 0.00 | 25.78 | 0.23 | 0.01 | 26.92 | 0.42 | 0.02 | 27.42 | 0.00 | 0.00 | 26.85 | 0.20 | 0.01 | 25.54 | 0.17 | 0.01 |
| SP6890 | 40.00 | 0.00 | 0.00 | 37.22 | 3.93 | 0.11 | 37.92 | 2.78 | 0.07 | 37.24 | 2.95 | 0.08 | 32.89 | 0.13 | 0.00 | 38.51 | 2.58 | 0.07 | 36.80 | 2.83 | 0.08 |
| SP6621 | 30.63 | 0.37 | 0.01 | 30.31 | 0.12 | 0.00 | 28.94 | 0.40 | 0.01 | 29.34 | 0.26 | 0.01 | 28.29 | 0.15 | 0.01 | 30.21 | 0.18 | 0.01 | 28.99 | 0.38 | 0.01 |
| SP8346 | 34.78 | 0.08 | 0.00 | 34.95 | 0.09 | 0.00 | 32.07 | 0.75 | 0.02 | 36.35 | 2.99 | 0.08 | 28.09 | 0.08 | 0.00 | 34.69 | 0.59 | 0.02 | 33.05 | 0.62 | 0.02 |
| SP6814 | 40.00 | 0.00 | 0.00 | 40.00 | 0.00 | 0.00 | 31.34 | 0.38 | 0.01 | 39.82 | 0.51 | 0.01 | 28.31 | 0.01 | 0.00 | 40.00 | 0.00 | 0.00 | 35.73 | 0.83 | 0.02 |
| SP7203 | 40.00 | 0.00 | 0.00 | 40.00 | 0.00 | 0.00 | 38.50 | 1.69 | 0.04 | 40.00 | 0.00 | 0.00 | 32.76 | 0.67 | 0.02 | 38.36 | 2.85 | 0.07 | 35.69 | 0.98 | 0.03 |
| SP4687 | 37.50 | 2.24 | 0.06 | 34.08 | 0.32 | 0.01 | 31.71 | 0.49 | 0.02 | 35.04 | 0.88 | 0.02 | 29.15 | 1.47 | 0.05 | 35.49 | 1.00 | 0.03 | 33.43 | 1.19 | 0.04 |
| SP6881 | 36.81 | 1.16 | 0.03 | 35.24 | 1.07 | 0.03 | 31.00 | 0.42 | 0.01 | 34.59 | 0.69 | 0.02 | 28.32 | 0.81 | 0.03 | 35.79 | 1.87 | 0.05 | 32.28 | 1.16 | 0.04 |
| SP1523 | 38.94 | 1.83 | 0.05 | 35.09 | 0.61 | 0.02 | 32.24 | 2.73 | 0.08 | 38.87 | 2.10 | 0.05 | 29.76 | 0.32 | 0.01 | 34.29 | 2.74 | 0.08 | 34.67 | 1.53 | 0.04 |
| SP0369 | 40.00 | 0.00 | 0.00 | 40.00 | 0.00 | 0.00 | 35.06 | 1.86 | 0.05 | 38.35 | 2.38 | 0.06 | 31.44 | 1.51 | 0.05 | 36.95 | 0.19 | 0.01 | 35.21 | 1.29 | 0.04 |
| SP8570 | 31.70 | 0.28 | 0.01 | 32.14 | 0.12 | 0.00 | 31.68 | 0.46 | 0.01 | 32.05 | 0.28 | 0.01 | 27.56 | 0.39 | 0.01 | 32.14 | 0.26 | 0.01 | 31.84 | 0.68 | 0.02 |
| SP0004 | 34.94 | 0.97 | 0.03 | 37.65 | 3.32 | 0.09 | 37.06 | 3.26 | 0.09 | 37.89 | 2.94 | 0.08 | 31.21 | 1.10 | 0.04 | 36.09 | 3.39 | 0.09 | 37.63 | 2.03 | 0.05 |
| SP0015 | 30.40 | 0.74 | 0.02 | 30.33 | 0.30 | 0.01 | 29.71 | 0.45 | 0.02 | 29.71 | 0.30 | 0.01 | 29.15 | 0.11 | 0.00 | 30.00 | 0.20 | 0.01 | 30.71 | 0.72 | 0.02 |
| SP0016 | 30.01 | 0.36 | 0.01 | 30.62 | 0.32 | 0.01 | 29.00 | 0.38 | 0.01 | 29.76 | 0.32 | 0.01 | 26.78 | 0.10 | 0.00 | 30.31 | 1.02 | 0.03 | 30.06 | 0.39 | 0.01 |
| SP0021 | 40.00 | 0.00 | 0.00 | 40.00 | 0.00 | 0.00 | 33.19 | 1.12 | 0.03 | 38.73 | 2.36 | 0.06 | 26.13 | 0.20 | 0.01 | 37.40 | 1.68 | 0.04 | 34.64 | 0.78 | 0.02 |
| SP0024 | 34.87 | 1.06 | 0.03 | 31.87 | 0.14 | 0.00 | 32.67 | 1.19 | 0.04 | 33.22 | 1.05 | 0.03 | 28.15 | 0.72 | 0.03 | 33.50 | 1.30 | 0.04 | 32.75 | 0.45 | 0.01 |
| SP0038 | 27.40 | 0.06 | 0.00 | 27.39 | 0.16 | 0.01 | 26.33 | 0.90 | 0.03 | 27.00 | 0.20 | 0.01 | 27.00 | 0.00 | 0.00 | 26.96 | 0.35 | 0.01 | 27.62 | 0.22 | 0.01 |
| SP0078 | 40.00 | 0.00 | 0.00 | 40.00 | 0.00 | 0.00 | 39.67 | 0.94 | 0.02 | 40.00 | 0.00 | 0.00 | 31.32 | 0.03 | 0.00 | 40.00 | 0.00 | 0.00 | 35.87 | 0.95 | 0.03 |
| SP2800 | 33.30 | 0.52 | 0.02 | 32.80 | 0.79 | 0.02 | 32.78 | 0.43 | 0.01 | 33.05 | 0.72 | 0.02 | 29.38 | 0.31 | 0.01 | 33.40 | 0.87 | 0.03 | 32.97 | 0.78 | 0.02 |
| SP3819 | 36.23 | 3.30 | 0.09 | 40.00 | 0.00 | 0.00 | 35.22 | 1.41 | 0.04 | 36.40 | 3.08 | 0.08 | 31.08 | 0.29 | 0.01 | 34.07 | 0.59 | 0.02 | 34.34 | 0.81 | 0.02 |
| SP6058 | 38.29 | 2.96 | 0.08 | 37.59 | 3.41 | 0.09 | 32.84 | 0.59 | 0.02 | 36.90 | 2.80 | 0.08 | 28.74 | 0.42 | 0.01 | 37.49 | 0.58 | 0.02 | 34.99 | 1.03 | 0.03 |
| SP7238 | 40.00 | 0.00 | 0.00 | 40.00 | 0.00 | 0.00 | 33.61 | 0.54 | 0.02 | 40.00 | 0.00 | 0.00 | 29.53 | 0.69 | 0.02 | 40.00 | 0.00 | 0.00 | 36.26 | 1.00 | 0.03 |
| SP9991 | 40.00 | 0.00 | 0.00 | 40.00 | 0.00 | 0.00 | 33.59 | 0.65 | 0.02 | 39.71 | 0.81 | 0.02 | 28.90 | 0.48 | 0.02 | 37.36 | 0.26 | 0.01 | 35.78 | 1.45 | 0.04 |
| Median CV | 0.01 | | | | | | | | | | | | | | | | | | | | |

SD; standard deviation, CV; coefficient of variation
